# Late recurrence of a tumor of Ewing’s sarcoma family of tumors: report of a case

**DOI:** 10.1186/s40792-015-0037-1

**Published:** 2015-04-24

**Authors:** Takamasa Yun, Hidemi Suzuki, Teruaki Mizobuchi, Yuichi Sakairi, Kaoru Nagato, Takahiro Nakajima, Takekazu Iwata, Shigetoshi Yoshida, Yukio Nakatani, Ichiro Yoshino

**Affiliations:** Department of General Thoracic Surgery, Chiba University Graduate School of Medicine, 1-8-1, Inohana, Chuo-ku, Chiba, 260-8670 Japan; Department of Pathology, Chiba University Graduate School of Medicine, 1-8-1, Inohana, Chuo-ku, Chiba, 260-8670 Japan

**Keywords:** Ewing’s sarcoma family of tumors, Primitive neuroectodermal tumor, Late recurrence

## Abstract

A 27-year-old female presented with a history of a right chest wall tumor at 3 years of age. At that time, the tumor was surgically resected and diagnosed as Ewing’s sarcoma (EWS), and postoperative chemoradiotherapy was administered. The patient remained disease-free for 25 years. At age 27, chest computed tomography revealed a mass adjacent to the anterolateral thoracic wall. After surgery, the diagnosis was primitive neuroectodermal tumor (PNET). She died of the disease 10 months later. PNET and EWS were integrated into a single item in the 2002 WHO classification; thus, they are considered clinically and pathologically identical. The morphologic, immunohistochemical, and molecular biological characteristics of both specimens showed that the second tumor was a local recurrence of Ewing’s sarcoma family of tumors (ESFT). Our case is the longest duration local recurrence reported. Long-term recurrences of ESFT and patients with recurrent ESFT have a poor prognosis; thus, long-term follow-up is necessary.

## Background

Tumors of Ewing’s sarcoma family of tumors (ESFT) are malignant tumors that originate in the central and peripheral nervous systems and soft tissues in children and adolescents. The prognosis is generally poor. However, recent treatment outcomes of ESFT have improved with the use of multimodal therapy [1]. Advances in molecular biology have revealed common chromosomal translocations such as EWS-FLI-1 among Ewing’s sarcoma (Ewing’s sarcoma of the bone and extraosseous Ewing’s sarcoma) and related diseases, such as primitive neuroectodermal tumor (PNET) and Askin’s tumor [2].

PNET and Ewing’s sarcoma (EWS) were integrated into a single item in the 2002 WHO classification; thus, they are considered clinically and pathologically identical. Late relapses are seldom described. We report a patient with an ESFT who had a recurrence 25 years after initial surgery and postoperative chemoradiation.

## Case presentation

**Figure 1:**
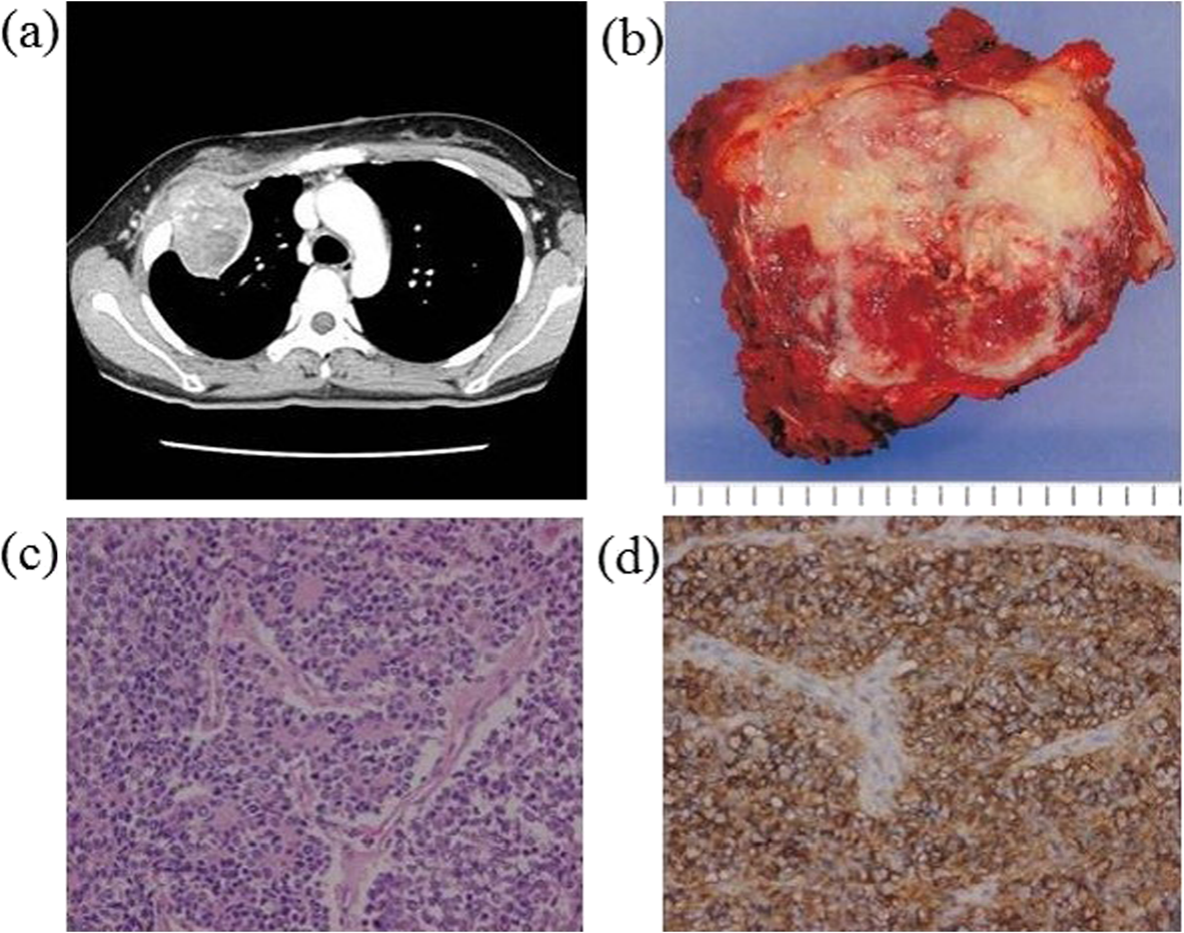
Diagnostic results at 3 years of age. **(a)** Thoracic magnetic resonance imaging at the time of initial surgery. **(b)** Macroscopic findings of the resected specimen at the time of initial surgery. **(c)** Sheets of small round cells with rosette formation (hematoxylin-eosin, ×400). **(d)** Immunohistochemical staining for CD99 antibody (×200). The tumor cell membrane stained positively for CD99.

A patient presented to us with a remote history of treatment for a right chest wall tumor. At 3 years of age, she was noted to have a very large chest wall tumor in the right thoracic cavity (Figure 1a) without distant metastases; she subsequently underwent surgical resection. The specimen revealed malignant small round cells with rosette formation and was positive for CD99 (MIC2 gene product) by immunohistochemical staining, consistent with a diagnosis of EWS (Figure 1b,c,d). The patient was administered three cycles of postoperative chemotherapy that consisted of vincristine, cyclophosphamide, adriamycin, and actinomycin-D, as well as local irradiation with a total dose of 40 Gy. Her postoperative course was uneventful, and she remained disease-free for 25 years.

**Figure 2:**
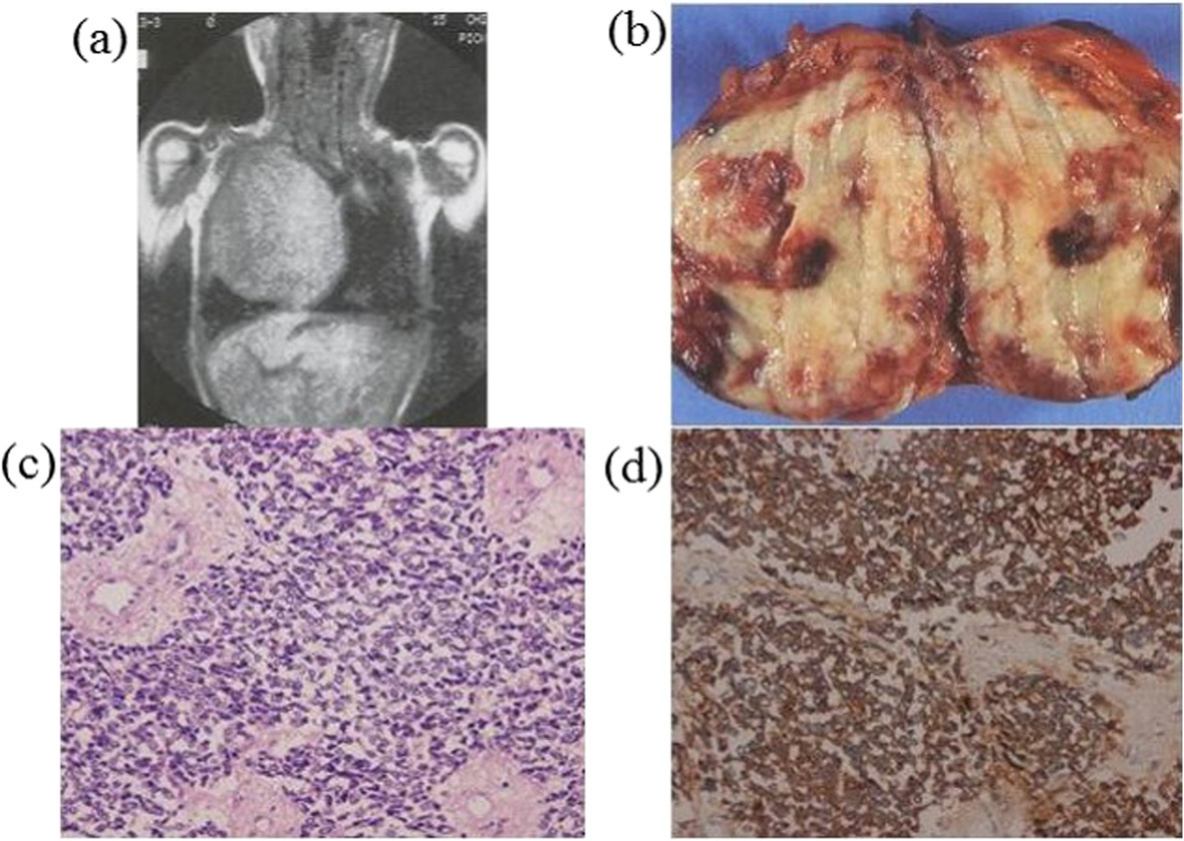
Diagnostic results at 27 years of age. **(a)** Chest computed tomography findings at the time of recurrence. A well-defined round mass is seen on the right upper lobe. **(b)** Resected specimen at the time of recurrence. The surgical specimen is a 55 × 40 × 40-mm soft tumor. The surface is smooth. The cut surface demonstrates a yellow-white, solid component with hemorrhage and focal necrosis. **(c)** Histopathological findings (hematoxylin-eosin, ×200). The tumor was composed of small round tumor cells that showed a rosette-like arrangement around the vessels. **(d)** Immunohistochemical staining for CD99 (×200). The tumor cell membrane stained positively for CD99.

At 27 years of age, she was referred to our institution with the chief complaint of chest pain. Her chest X-ray demonstrated a well-defined, round mass in the right upper lung field. A computed tomography (CT) scan of the chest revealed a distinct mass adjacent to the anterolateral thoracic wall (Figure 2a), and metastatic workup was negative. Physical examination was unremarkable. Hematology and blood chemistry values were within the normal ranges, and tumor markers (carcinoembryonic antigen, neuron-specific enolase, squamous cell carcinoma-related antigen, and α-fetoprotein) were lower than the standard values.

As either radiation-induced sarcoma or a local recurrence of EWS was suspected, we performed extirpation of the tumor and chest wall (the left second to the fourth ribs), partial resection of the right lung, and reconstruction of the chest wall (Figure 2b). Intraoperative blood loss was 800 g, and the operation took 4 h and 51 min.

Pathologic findings were identical to those of the original chest wall tumor, with small round cells and extensive necrosis (Figure 2c). The specimen stained positively with antibodies to CD99 (Figure 2d). Immunohistochemical and histochemical staining showed that the tumor was positive for chromogranin, synaptophysin, and neuron-specific enolase and negative for thyroid transcription factor-1, myogenin, and desmin. Fluorescence *in situ* hybridization analysis using a EWSR1 break-apart probe identified the rearranged EWSR1 gene in both cases. Thus, histopathologically, the tumor was diagnosed as a PNET. Four cycles of VDC (vincristine, doxorubicin, cyclophosphamide) chemotherapy and two cycles of IE (ifosfamide, etoposide) and high-dose L-PAM (melphalan) and radiotherapy of left thigh bone for a total dose of 30 Gy was administered after the second operation. She had recurrences in the right chest wall and upper middle lobe and metastases in the skull, rib, vertebrae, ilium, and thigh bone. She died of carcinomatous lymphangiosis 10 months after the second surgery.

## Discussion

In 1921, James Ewing reported a rare type of sarcoma in children, adolescents, and young adults that is a highly malignant tumor of the bone and soft tissue, composed of small round cells [3]. Separately, Stout et al. reported in 1918 that a PNET is an ulnar nerve-derived small round cell tumor with rosette formation that develops in soft tissues in young individuals [4]. The first small round cell tumor of chest wall origin was reported in a pediatric patient by Askin, and these tumors are referred to as Askin tumors [5]. An Askin tumor is a PNET that develops from the soft tissues of the chest wall in childhood. At that time, our case might have been termed an Askin tumor.

Pathologically, EWS/PNET is composed of small, round, uniform cells with characteristic small-sized rosette formation. These tumors, both with a poor prognosis, were described as separate entities in the small round cell tumor group in the second version of the WHO classification published in 1993. However, with recent improvements in molecular biology, a translocation (11; 22) (q24; q12) specific to both EWS and PNET has been identified, and the EWS-FLI-1 fused gene was identified at the cleavage site of this translocation. They therefore were subsequently integrated into a single item (Ewing’s sarcoma/PNET) in the WHO classification, published in 2007 [6]. The ESFT includes EWS of the bone, extraosseous EWS, and PNET of the bone or soft tissue.

The most useful immunohistologic reagent for the diagnosis of ESFT is the monoclonal antibody CD99, which recognizes a cell surface protein. Specimens stain strongly positive with the CD99 antibody in more than 90% to 95% of cases of reported ESFT.

Chest wall ESFT tumors are rare, have a high rate of local recurrence, and frequently are metastatic at presentation. In a report of 84 thoracic PNET cases by Biswas et al., patient age ranged from 3 to 40 years (mean age = 15 years) and tumor diameter ranged from 1.6 to 20.0 cm (mean diameter = 9.0 cm). It occurred most commonly in the ribs (54%), the scapula (25%), and the chest wall (14%), with a male predominance (70%). Twenty-seven (32%) patients had metastatic disease at diagnosis. An independent prognostic factor for PNET is the presence of metastatic disease [7]. The median relapse-free interval (RFI) after treatment was 17 months (range, 5 to 90 months). In contrast to the dramatic improvement in survival for ESFT, the prognosis after recurrent disease remains poor, especially for patients with a RFI of less than 24 months [8].

The morphologic, immunohistochemical, and molecular biological characteristics from both specimens supported that the second tumor was a local recurrence of an ESFT. Several prior studies established that the longest time until recurrence of ESFT was 19 years [9]. Our case demonstrates the possibility of very late local recurrence of ESFT.

We report a case of locally recurrent ESFT 25 years after initial treatment. To our knowledge, no case with such late local relapse as our case has been reported to date.

## Conclusions

We report a rare case of chest wall ESFT in a 27-year-old female. A right thoracic recurrence was surgically resected 25 years after the primary tumor, but the patient died of the disease 10 months after surgery. Our case suggests that patients with ESFT should undergo lifetime surveillance. Generally, it is difficult to detect recurrence from clinical symptom of the patients. Patients should be under observation using periodical CT, bone scintigraphy, or PET periodically.

## Consent

Written informed consent was obtained from the patient for publication of this case report and any accompanying images.
